# Supplementary material for: Iterative improvement in the automatic modular design of robot swarms
Source: PeerJ Comput Sci. 2020 Dec 7;6:e322. doi: 10.7717/peerj-cs.322 (PMC7924708; doi:10.7717/peerj-cs.322)
Supplement: Supplemental Information 3 [file peerj-cs-06-322-s003.zip › argos3/doc/api/standalone/a00533_source.html]

ARGoS: plugins/robots/generic/simulator/light\_default\_sensor.h Source File


- Main Page
- Related Pages
- Namespaces
- Classes
- Files

- File List
- File Members

# plugins/robots/generic/simulator/light\_default\_sensor.h

Go to the documentation of this file.

```
00001 
00007 #ifndef LIGHT_DEFAULT_SENSOR_H
00008 #define LIGHT_DEFAULT_SENSOR_H
00009 
00010 #include <string>
00011 #include <map>
00012 
00013 namespace argos {
00014    class CLightDefaultSensor;
00015    class CLightSensorEquippedEntity;
00016 }
00017 
00018 #include <argos3/plugins/robots/generic/control_interface/ci_light_sensor.h>
00019 #include <argos3/core/utility/math/range.h>
00020 #include <argos3/core/utility/math/rng.h>
00021 #include <argos3/core/simulator/space/space.h>
00022 #include <argos3/core/simulator/sensor.h>
00023 
00024 namespace argos {
00025 
00026    class CLightDefaultSensor : public CSimulatedSensor,
00027                                    public CCI_LightSensor {
00028 
00029    public:
00030 
00031       CLightDefaultSensor();
00032 
00033       virtual ~CLightDefaultSensor() {}
00034 
00035       virtual void SetRobot(CComposableEntity& c_entity);
00036 
00037       virtual void Init(TConfigurationNode& t_tree);
00038 
00039       virtual void Update();
00040 
00041       virtual void Reset();
00042 
00052       virtual Real CalculateReading(Real f_distance, Real f_intensity);
00053 
00054    protected:
00055 
00057       CLightSensorEquippedEntity* m_pcLightEntity;
00058 
00060       CControllableEntity* m_pcControllableEntity;
00061 
00063       bool m_bShowRays;
00064 
00066       CRandom::CRNG* m_pcRNG;
00067 
00069       bool m_bAddNoise;
00070 
00072       CRange<Real> m_cNoiseRange;
00073 
00075       CSpace& m_cSpace;
00076    };
00077 
00078 }
00079 
00080 #endif
```

---

Generated on 10 Jul 2018 for ARGoS by 
 1.6.1
